# Counteracting Action of Curcumin on High Glucose-Induced Chemoresistance in Hepatic Carcinoma Cells

**DOI:** 10.3389/fonc.2021.738961

**Published:** 2021-10-06

**Authors:** Vivek Kumar Soni, Arundhati Mehta, Yashwant Kumar Ratre, Vikas Chandra, Dhananjay Shukla, Ajay Kumar, Naveen Kumar Vishvakarma

**Affiliations:** ^1^ Department of Biotechnology, Guru Ghasidas Vishwavidyalaya, Bilaspur, India; ^2^ Department of Zoology, Banaras Hindu University, Varanasi, India

**Keywords:** curcumin, chemoresistance, glucose, hepatic cancer, metabolism

## Abstract

Along with direct anticancer activity, curcumin hinders the onset of chemoresistance. Among many, high glucose condition is a key driving factor for chemoresistance. However, the ability of curcumin remains unexplored against high glucose-induced chemoresistance. Moreover, chemoresistance is major hindrance in effective clinical management of liver cancer. Using hepatic carcinoma HepG2 cells, the present investigation demonstrates that high glucose induces chemoresistance, which is averted by the simultaneous presence of curcumin. Curcumin obviated the hyperglycemia-induced modulations like elevated glucose consumption, lactate production, and extracellular acidification, and diminished nitric oxide and reactive oxygen species (ROS) production. Modulated molecular regulators are suggested to play a crucial role as curcumin pretreatment also prevented the onset of chemoresistance by high glucose. High glucose instigated suppression in the intracellular accumulation of anticancer drug doxorubicin and drug-induced chromatin compactness along with declined expression of drug efflux pump MDR-1 and transcription factors and signal transducers governing the survival, aggressiveness, and apoptotic cell death (p53, HIF-1α, mTOR, MYC, STAT3). Curcumin alleviated the suppression of drug retention and nuclear condensation along with hindering the high glucose-induced alterations in transcription factors and signal transducers. High glucose-driven resistance in cancer cells was associated with elevated expression of metabolic enzymes HKII, PFK1, GAPDH, PKM2, LDH-A, IDH3A, and FASN. Metabolite transporters and receptors (GLUT-1, MCT-1, MCT-4, and HCAR-1) were also found upregulated in high glucose exposed HepG2 cells. Curcumin inhibited the elevated expression of these enzymes, transporters, and receptors in cancer cells. Curcumin also uplifted the SDH expression, which was inhibited in high glucose condition. Taken together, the findings of the present investigation first time demonstrate the ability of curcumin against high glucose-induced chemoresistance, along with its molecular mechanism. This will have implication in therapeutic management of malignancies in diabetic conditions.

## Introduction

Chemoresistance is one of the major hurdles in the efficacious outcome of treatment strategies against various malignancies including liver cancer (1, 2). Various factors have been indicated to favor the onset of chemoresistance (1, 3). The metabolic influences in instigating and maintaining the resistant phenotype have been demonstrated (1, 3, 4). The “aerobic glycolysis” (Warburg effect) also serves as a landscape for the selection of aggressive cancer cells (5). Moreover, a hyperglycemic condition in the extracellular milieu has been shown to confer advantages to cancer cells including the onset of chemoresistance (4, 6–9). In a comparison of meta-analyses, Li et al. (9) reported highest relative risk for liver cancer in diabetic patients (9). Moreover, diabetes has been linked with resistance to chemotherapy (10–13). The survival benefits triggered by high glucose levels may vary based on the stage of cancer progression or metabolic capabilities (8). Decreased susceptibility towards induction of apoptosis through modulated mitochondria-dependent pathway under hyperglycemic conditions promotes chemoresistance in cancer cells (4). Accelerated glycolysis favored by a high level of glucose can also contribute to the modulated metabolite levels and biophysical constitution of the tumor microenvironment (5, 8). A high rate of glycolysis leads to extracellular accumulation of lactate, and tumor acidosis; both of these endorse chemoresistant phenotype (5, 14). Strategies targeting cancer metabolism and associated adaptations are expected to have therapeutic benefits against chemoresistance (1, 14). Many inhibitors of plant origin have shown their promises in the modulation of cancer metabolism (14–16). Curcumin, the bioactive component and yellow pigment of turmeric, has proven antineoplastic activity and the ability to affect the metabolism of malignant cells (14, 16–18). Along with metabolic modulatory potential, curcumin has benefits as adjuvant in cancer therapy (14, 16, 19).

Curcumin has a proven ability to counter chemoresistance in cancer cells (14, 19, 20). The ability of curcumin to modulate the regulatory networks governing the balance of cell survival and induction of cell death is well established (20, 21). Curcumin has been demonstrated to amend the expression of molecules central to chemoresistance including members of the ABC drug efflux transporter family (14, 17, 22). Curcumin modulates the cancer metabolism and bio-physiological composition of the extracellular milieu culminating in the induction of cell death and retardation in disease progression (17). Metabolic alterations and suppression of receptor-mediated signaling were suggested to provide chemosensitization of cancer cells by curcumin (14, 19, 20). The previous investigation on hepatic carcinoma cells demonstrated that curcumin can thwart lactate-induced chemoresistance (14). Interestingly, curcumin can be exploited to provide health benefits in diabetes mellitus owing to its antioxidant and anti-inflammatory capabilities (16). In renal tubular epithelial cells, curcumin was also shown to obviate high glucose-induced epithelial-to-mesenchymal transition (EMT) (23). High glucose conditions can aggravate the invasion and migration (24), while curcumin can impede the metastatic events in a variety of malignancies (18, 21). Antineoplastic potential, metabolic modulatory ability, and chemosensitizing property along with safety investigations (15, 16, 20, 21, 25, 26) provide an edge to curcumin over other phytochemicals. However, the potential of curcumin against high glucose-induced chemoresistance remains undefined. Among malignancies, risk of liver cancer is relatively high in hyperglycemia (9); and it also serves as good model for investigation on chemoresistance (27). Therefore, the present investigation was intended to explore the high glucose to induce chemoresistance in hepatic carcinoma cells and the counteracting ability of curcumin. Attempts were also made to observe the effect of curcumin on hyperglycemia-associated manifestation on cancer cells along with elucidation of the underlying mechanism.

## Materials and Methods

### Reagents and Cancer Cells

All reagents used were of tissue culture or analytical grade. Tissue culture medium DMEM was purchased from HiMedia (Bombay, India). All culture media were supplemented with 20 mg/ml gentamycin, 0.1 mg/ml streptomycin, 100 IU penicillin purchased from HiMedia (Bombay, India), and 10% fetal bovine serum from HiMedia (Bombay, India), henceforth, referred to as complete medium. Medium without serum was designated as an incomplete medium. Curcumin used was procured from HiMedia (Bombay, India) and was dissolved in DMSO to prepare the stock. The stock of curcumin was further diluted in a complete medium to obtain a working solution (17). Anticancer Drugs doxorubicin and methotrexate were procured from HiMedia (Bombay, India). Authenticated hepatic carcinoma HepG2cells were procured from cell repository at National Centre for Cell Science (NCCS) (Pune, India), Government of India. The cell line was maintained *in vitro* in the passage as well as in cryopreserved form. The maximum passage number of cells used was less than 25; and the passage number difference in any two experiments in this investigation was less than five.

### Assay for Cancer Cell Survival and Calculation of IC_50_

The viability of cancer cells was enumerated by a standard trypan blue dye exclusion assay. The survival of cancer cells was assayed according to a method described earlier (28). Briefly, cells incubated in 96-well plate, with culture condition indicated in the results, were stained with 0.5% (w/v) crystal violet solution containing 20% (v/v) methanol. After washing with PBS, 200 µl methanol was added to each well and incubated at room temperature in rocking condition for 20 min. Absorbance of control (*A_control_*) and treated (*A_treated_*) groups were read at 590 nm on a Multiscan EX mirotitre plate reader. Relative survival was calculated using a formula as follows:


$$\%\;relative\;survival=\frac{A_{treated}}{A_{control}}\times 100$$

For calculation of IC_50_, cells were treated with various concentrations of anticancer drugs (0–20 mM) in conditions indicated in the results. After 24 h of incubation, the survival of cancer cells was estimated. IC_50_ was calculated by a linear interpolation method using the following formula:


$$IC_{50}=\left[\left(\frac{50-A}{B-A}\right)\times (D-C)\right]+C$$

where A = the first point on the curve that is less than 50% inhibition, B = the first point on the curve that is greater than or equal to 50% inhibition, C = the concentration of drug that gives A% inhibition, and D = the concentration of drug that gives B% inhibition.

### Estimation of Metabolic Activity

Metabolic activity of cells was estimated using MTT assay (14). Cancer cells (1 × 10^5^/ml) were seeded in a 96-well tissue culture plate in a complete medium with indicated concentrations of curcumin and/or standard anticancer drugs or additional glucose, as described in *Results*. Cell survival was measured by standard MTT assay. MTT [3-(4,5-dimethylthiazol-2yl)-2,5-diphenyl tetrazolium bromide] was dissolved in PBS at a concentration of 5 mg/ml of MTT solution. A 50 µl of the solution was added to each well of the 96-well culture plate containing cells in 200 µl medium and incubated at 37°C for 4 h for the formation of dark blue formazan crystals. The medium was then carefully removed without disturbing the crystals. DMSO (50 µl) was added to each well and mixed thoroughly to dissolve the formazan crystals. The plates were then read on a Multiscan EX microplate reader (Thermo Scientific) at a wavelength of 540 nm. Metabolic activities of cells are presented as relative to control.

### Enumeration of Dead Cell

Dead cell percentage was enumerated with the method described earlier (29). Briefly, cancer cells were tissue culture plate in complete medium and were treated as given in results. Cells were then harvested using trypsin-EDTA, and the number of live (*N_Viable_*) and death cells (*N_Dead_*) populations was enumerated by hemocytometer and microscopy techniques using the trypan blue dye exclusion method. Death cell percent was calculated using a standard formula.


$$Dead\;cells\;(\%)=\frac{N_{Dead}}{N_{Viable}+N_{Dead}}\times 100$$

### Morphological Evaluation of Apoptotic Cells by Wright-Giemsa Staining

The apoptotic cell population was enumerated by a method described earlier (17). Air-dried and methanol-fixed smear of cell suspension was stained with Wright-Giemsa staining solution, mounted in glycerin, and analyzed under a light microscope (Leica, India). Morphological features associated with apoptotic cells include the contracted cell bodies, condensed, uniformly circumscribed, and densely stained chromatin, and membrane-bound apoptotic bodies containing one or more nuclear fragments. The percentage of apoptotic cells was determined by counting at least three hundred cells in at least three separate microscopic fields.

### Estimation of Nitrite

The concentration of stable nitrite NO_2_, the end product from NO generation, was determined in the cell-free culture supernatant by the method described earlier (30) with slight modification based on the Griess reaction. Culture supernatants were incubated with an equal volume of Griess reagent [one part of 1% (w/v) sulfanilamide in 2.5% H3PO4 plus one part of 0.1% (w/v) naphthyl-ethylene-diamine dihydrochloride; two parts being mixed within 12 h of use and kept chilled at room temperature for 10 min in a 96-well Microtiter plate]. The absorbance at 540 nm was determined by an automatic ELISA plate reader (Thermo Scientific). Nitrite content was quantified by extrapolation from a standard curve of NaNO_2_ in each experiment. In all the experiments, nitrite content in the wells containing medium without cells was also measured and subtracted.

### Quantification of Glucose

Glucose content was measured using a commercial kit from Beacon Diagnostics Pvt. Ltd, India, based on the conversion of glucose to H_2_O_2_ by the action of glucose oxidase and final estimation of the generated H_2_O_2_ by converting it into a colored red quinone product by the action of peroxidase (14). Briefly, 10 ml of fluid sample was mixed with 1 ml of working reagent containing phosphate buffer (pH 7.4), phenol, glucose-oxidase, peroxidase, and 4-Aminoantipyrine and was incubated for 10 min at 37˚C. The final reading was taken at 505 nm. Glucose content is expressed as mM. To determine the glucose consumption, the differences of values for culture supernatant and uninoculated media were determined.

### Estimation of Lactate

Lactate concentration was measured using an enzymatic colorimetric method (Spinreact, Granada, Spain) (17). Briefly, 1 ml sample was diluted in 200 ml 50 mM PIPES (pH 7.5) containing 4-chlorophenol (4 mM), lactate oxidase (800 U/L), peroxidase (2,000 U/L), and 4-aminophenazone (0.4 mM), followed by incubation for 10 min at room temperature, and measurement of absorbance at 505 nm was recorded. Lactate concentration was expressed in mM.

### Extracellular pH

Culture supernatants of HepG2 cells (1 × 10^5^/ml) treated with curcumin in a medium containing standard or additional glucose concentration were collected after 24 h of incubation. The pH level of cell-free culture supernatants was measured using a probe of pH meter.

### Estimation of Percent DNA Fragmentation

Induction of apoptotic mode of cell death in cells was also confirmed by quantitative determination of DNA fragmentation (29). Tumor cells were lysed in 0.5 ml lysis buffer (0.2% v/v TritonX-100 in Tris-EDTA buffer, pH 7.4) and centrifuged (13,000 g at 4°C for 10 min) to separate intact and fragmented DNA in pellet and supernatant in tubes. Trichloroacetic acid (0.5 ml of 25%) was added to each tube and mixed thoroughly using a vortex. Tubes were kept overnight at 4°C for DNA precipitation. After centrifugation (13,000 g at 4°C for 10 min), pellets were mixed with 80 µl of 5% trichloroacetic acid. DNA was hydrolyzed by heating at 90°C for 15 min, mixed with 160 µl diphenylamine (DPA) reagent prepared by dissolving the 150 mg diphenylamine in a mixture of 10 ml glacial acetic acid, 150 ml concentrated H_2_SO_4_, and 50 ml of acetaldehyde solution. A blank was included containing 80 µl of 5% trichloroacetic acid. The reaction mixture in tubes was allowed to develop color overnight at room temperature. Absorbance was measured at 600 nm in a Multiscan EX microplate reader (Thermo Scientific). Percent DNA fragmentation was calculated by putting the values of absorbance for a tube containing fragmented (*A_frag_*) or intact (*A_int_*) DNA in the following formula:


$$DNA\;Fragmentation\;(\%)=\frac{A_{frag}}{A_{frag}+A_{\mathrm{int}}}\times 100$$

### DAPI Staining for Nuclear Morphology/Chromatin Condensation

Cell nuclear morphology was evaluated by fluorescence microscopy following DAPI staining described earlier (31) with slight modification. Briefly, cells were washed with PBS and fixed with ice-cold methanol. Fixed cells were then stained with DAPI (300 nM) for 20 min at 4°C in the dark. Stained cells were washed briefly with PBS and were observed under a fluorescence microscope (Leica, India) using a 358 nm excitation and 460 nm emission fluorescent filter. Images were captured and transformed for Sobel edge detection. Images were then analyzed for mean intensity and standard deviation of DAPI intensities of the pixels in the image of individual nuclei using ImageJ software. The coefficient of variation was obtained by dividing the standard deviation by mean as a quantitative measure of chromatin condensation (32).

### PI Staining of Dead Cells

Compromised membrane integrity accompanies cell death which can be detected by PI uptake by dying cells. The method described earlier was used with slight modification (33). Briefly, cells were washed with PBS and then stained with PI (10 nM) for 30 min at 4°C in the dark. Stained cells were then washed briefly with PBS and immediately observed under a fluorescence microscope (Leica, India) using a 535 nm excitation and 617 nm emission fluorescent filter. Red fluorescence (PI-positive) in cells indicated the compromised membrane integrity and death. Cells with and without fluorescence were enumerated to calculate the percent PI-positive cells.

### Drug-Uptake Assay

Drug uptake was evaluated by fluorescence microscopy following the doxorubicin staining described earlier (34) with slight modification. HepG2 cells (1 × 10^5^/ml) were seeded in the 12-well culture plate in a medium containing the concentration of glucose and curcumin as indicated in the results. After incubation of 24 h at 37°C in a CO_2_ incubator, culture supernatant was removed; cells were rinsed twice with sterile PBS. Doxorubicin (25 μM) was then added to each well and incubated for 15 min at RT. After washing with sterile PBS, cells were observed under a fluorescence microscope (Leica, India) using appropriate excitation and emission filters. Images were captured, and doxorubicin fluorescence intensity was quantified by ImageJ software.

### Quantification of ROS Generation

Level of ROS was evaluated by fluorescence microscopy following DCFDA staining described earlier (35) with slight modification. HepG2 cells (~1 × 10^5^/ml) were plated in the 12-well culture plate in a medium containing the indicated amount of glucose for 24 h. Cells were then washed with sterile PBS twice, and 10 μM DCFDA was added for 30 min at 37°C followed by stimulation with curcumin, doxorubicin, or both for 15 min. After washing, cells were observed under a fluorescence microscope (Leica, India) using a 535 nm excitation and 635 nm emission fluorescent filter. Images were captured and the intensity of the stain was quantified by ImageJ software.

### Reverse Transcriptase-PCR for Expression of mRNA

RT-PCR analysis for the expression of indicated genes, and β-actin was carried out according to a method described earlier (8). Primer sequences for various genes are shown in **Table 1**. PCR was performed for 15 min to make cDNA at 50°C. The amplification was carried out for 35 cycles with an initial denaturation at 94°C for 2 min followed by annealing (annealing temperature as per respective primer design) for 30 s and elongation at 72°C for 30 s. The samples were separated on an agarose gel (1%) containing ethidium bromide (0.3 mg/ml). Bands were visualized and analyzed on a UV-transilluminator (Biorad, Australia), and the intensity of bands was analyzed by ImageJ software. Band intensities of β-actin were used as a loading control. To comply with MIQE guidelines (36), invariable expression of β-actin was ensured by comparing the band intensities among experimental groups and obtaining coefficient of correlation not less than 0.95 between band intensity and amount of template used for PCR amplification (**Supplementary Figures 1** and **2**).

**Table 1:** Primer sequences for RT-PCR analysis.

S. No.	Gene	Primer Sequence
1	FASN	F-5’- TCGTGGGCTACAGCATGGT-3’ R- 5’-GCCCTCTGAAGTCGAAGAAGAA-3’
2	GAPDH	F -5’- ACGGATTTGGTCGTATTGGG-3’ R -4’-TGATTTTGGAGGGATCTCGC-3’
3	GLUT-1	F -5′- CTTTGTGGCCTTCTTTGAAGT-3′ R -5′- CCACACAGTTGCTCCACAT-3′
4	MYC	F -5′-CCTGGTGCTCCATGAGGAGAC-3 R -5′-CAGACTCTGACCTTTTGCCAGG-3′
5	HCAR-1	F-5’-AATTTGGCCGTGGCTGATTTC-3’ R-5’- ACCGTAAGGAACACGATGCTC-3’
6	HIF-1α	F-5’-TGAGCTCACATCTTGATAAAGCTTCT-3’ R-5’-GGGCTTTCAGATAAAAACAGTCCAT-3’
7	HKII	F -5’- GAGTTTGACCTGGATGTGGTTGC-3’ R -5’- CCTCCATGTAGCAGGCATTGCT-3’
8	IDH3A	F- 5’-TGCTGAGTTTGCCTTTGAGTATG-3’ R- 5’-CGCATGATGTTGGCTTTGTG-3’
9	LDH-A	F-5’-GGACAGTGCCTACGAGGTGAT-3’ R-5’-GGATGCACCCGCCTAAGG-3’
10	MCT-1	F-5’-CACTTAAAAATGCCACCAGCA-3’ R-5’-AGAGAAGCCGATGGAAATGA-3’
11	MCT-4	F-5’- GTTGGGTTTGGCACTCAACT -3’ R-5’- GAAGACAGGGCTACCTGCTG -3’
12	MDR-1	F-5’-GCTCATCGTTTGTCTACAGTTCGT-3’ R-5’-ACAATGACTCCATCATCGAAACC-3’
13	mTOR	F-5’-TTGGAATCTGAGTGCAGTGG-3’ R-5’- TTGGAATCTGAGTGCAGTGG-3’
14	p53	R -5’- CCTCAGCATCTTATCCGAGTGG-3’ F-5’- TGGATGGTGGTACAGTCAGAGC-3’
15	PFK1	F -5’-TACGACTTCTTTCGGCATGA-3’ R -5’-CTCCTCTCCCGGGTTGTAT-3’
16	PKM2	F -5′-TGCAATTATTTGAGGAACTCC-3′ R -5′-CACTGCAGCACTTGAAGGAG-3′
17	SDH	F- 5’-TGGTCATTCAGAGCACTACTTC-3’ R- 5’-AACTGTTGTCAAGGTCACGAA-3’
18	STAT3	F- 5’-CAAGCCTTTCCTGACAGAGG-3’ R -5’-TTGGAATCTGAGTGCAGTGG-3’
19	β-actin	F -5′- ACTCTTCCAGCCTTCCTTC-3′ R -5′- ATCTCCTTCTGCATCCTGTC-3′

### Western Blot Analysis

Expression level of proteins was estimated by western blot analysis (35). Protein content in cell lysates prepared with Triton-X 100 lysis buffer was determined by Bradford assay. Equal protein contents separated on SDS-PAGE were transferred on nitrocellulose membrane followed by their probing with specific primary antibodies. Membranes were then incubated with alkaline phosphatase conjugated secondary antibody. After washing to remove unbound antibodies, protein bands were detected by using BCIP/NBT solution. Membranes containing developed bands were then scanned, and band intensities were measured by ImageJ software. Intensities of band of specific proteins were compared with band intensities of β-actin (loading control).

### Statistical Analysis

All the experiments were conducted at least thrice in triplicate. The statistical significance of differences between test groups was analyzed by one-tailed or two-tailed *Student’s t-test* as appropriate. The difference was considered significant only when the *p*-value was less than 0.05 in compared groups.

## Results

### Hyperglycemia Augments the Survival of HepG2 Cells

**Figure 1:**
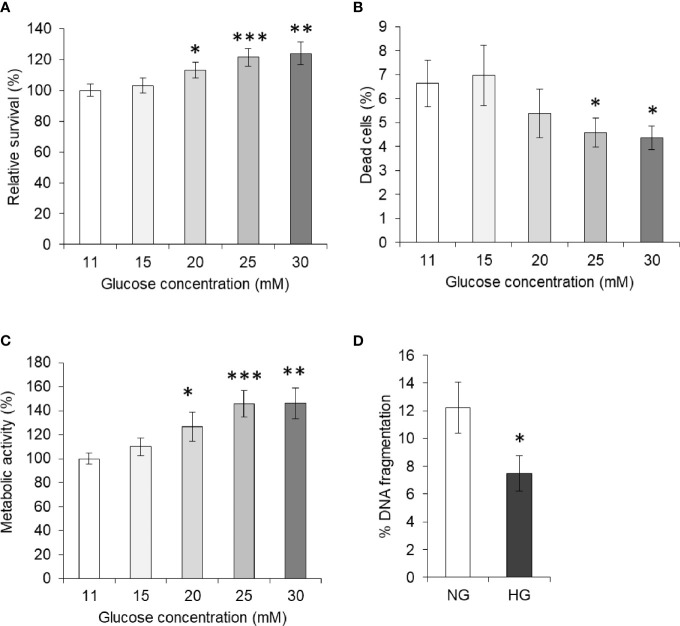
High glucose enhances cell survival. Survival of HepG2 cells incubated in medium containing standard (11 mM) or higher concentration of glucose was estimated **(A)**. Cells were also enumerated for live and dead cell population, and dead cell percentage was calculated **(B)**. Metabolic activity of cells incubated in medium containing increasing concentration of glucose was estimated using MTT assay **(C)**. DNA fragmentation of cells incubated in medium with normal glucose (NG) (11 mM) or High glucose (HG) (25 mM) was determined **(D)**. The values shown are Mean ± SD of three independent experiments conducted in triplicate. *p < 0.05, **p < 0.01, ***p < 0.001 *vs* values of cells incubated in medium with NG (11 mM).

To enumerate the effect of elevated levels of glucose on tumor cells’ survival, HepG2 (1×10^5^/ml) cells were incubated in complete medium alone having a standard concentration of glucose (11 mM) or containing an additional gradient of glucose concentration. After incubation for 24 h, cell survival was measured. Results are shown in **Figure 1A**. A concentration-dependent increase in the survival of HepG2 cells was observed. Statistically significant augmentation in cell survival was observed up to 25 mM concentration of glucose. The dead cell population was also enumerated using the trypan blue dye exclusion method and results are given in **Figure 1B**. A decline in the percent dead cell population was recorded with an increase in the glucose concentration. Metabolic activity of cells was also found to augment by additional glucose level in a concentration-dependent manner (**Figure 1C**). For augmentation of survival and metabolic activity or decline in the dead cell population, a peak statistically significant deviation was observed in media containing 25 mM glucose. Therefore, hereafter 25 mM concentration of glucose was termed as high glucose (HG) and was used in further experiments, otherwise stated. The standard concentration of glucose in media (11 mM) was used as normal glucose (NG) concentration.

### High Glucose Protects HepG2 Cells From DNA Fragmentation

In order to evaluate the effect of HG on the apoptotic parameter, HepG2 cells were incubated in a medium containing either NG or HG levels. After 24 h of incubation, cells were harvested and processed for determination of DNA fragmentation as per the method described in *Materials and Methods*. As indicated in **Figure 1D**, the DNA fragmentation level was found to be significantly lower in the cells incubated with HG concentration.

### Hyperglycemia Provokes Drug Resistance in Cancer Cells

**Figure 2:**
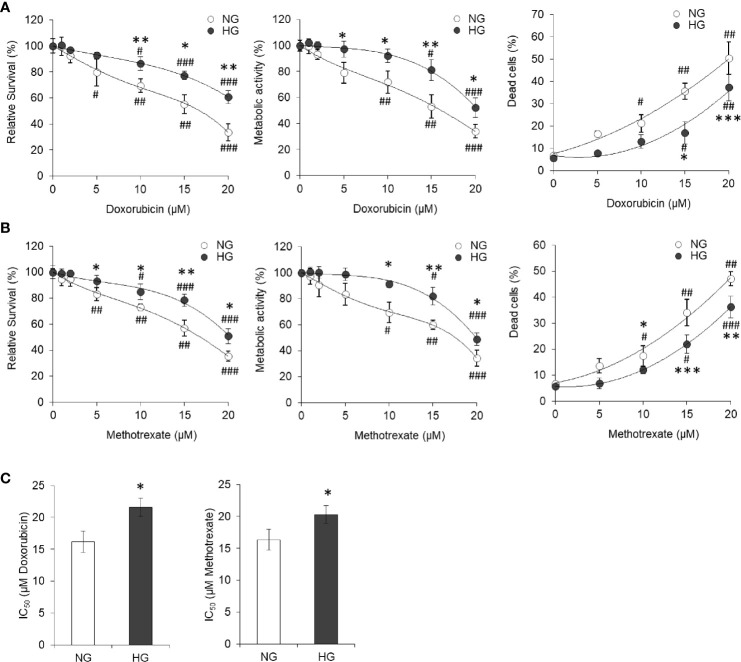
High glucose promotes the chemoresistance HepG2 cells. HepG2 cells incubated in NG or HG medium with increasing concentration of anticancer drugs doxorubicin **(A)** and methotrexate **(B)** for 24 h followed by estimation of cell survival (left panels), metabolic activity (middle panels), and enumeration of percentage of dead cells (right panels). IC_50_ was estimated for cells incubated in NG or HG medium for both the anticancer drugs doxorubicin and methotrexate **(C)**. The values shown are Mean ± SD of three independent experiments conducted in triplicate. *p < 0.05, **p < 0.01, ***p < 0.001 *vs* values of cells incubated in NG medium. ^#^ *p* < 0.05, ^##^ *p* < 0.01, ^###^ *p* < 0.001 *vs* values of cells incubated in medium without anticancer drugs.

For evaluation of the effect of elevated levels of glucose on tumor cells’ susceptibility towards cytotoxic action of standard chemotherapeutic drugs, HepG2 cells (1×10^5^/ml) were incubated for 24 h in a medium having NG or HG level and containing either doxorubicin or methotrexate in increasing concentration. After incubation, estimation of cell survival and metabolic activity and enumeration of the dead cell population were carried out using standard crystal violet assay, MTT assay, and trypan blue dye exclusion assay, respectively. Results are given in **Figures 2A, B**. Survival of cancer cells was found to decline with an increase in the concentration of anticancer drugs either doxorubicin or methotrexate. Observed decline of cell survival was less in cells incubated in HG medium. The survival of HepG2 cells incubated with anticancer drugs in HG-containing medium was significantly higher as compared to those incubated in NG medium containing the same concentration of either doxorubicin or methotrexate. Similar effect of anticancer drugs was observed on metabolic activity of HepG2 cells incubated in NG or HG medium. The HG was found to resist the increase in dead cell population with an increasing level of anticancer drugs as indicated in **Figures 2A, B**. Tumor cells treated with anticancer drugs in HG-containing medium had a significantly lower level of percent dead cell population as compared to those incubated in NG medium. Relative abundance of dead cells *versus* live cell was found to increase in anticancer drug–treated groups (**Supplementary Figure 3**). However, ratio of dead and live cells was significantly lower in the cells treated with anticancer drugs in HG medium. IC_50_ of both the anticancer drugs were estimated, and results are given in **Figure 2C**. HG level was found to increase the IC_50_ of both the drugs (doxorubicin and methotrexate) against HepG2 cells (**Figure 2C**).

**Figure 3:**
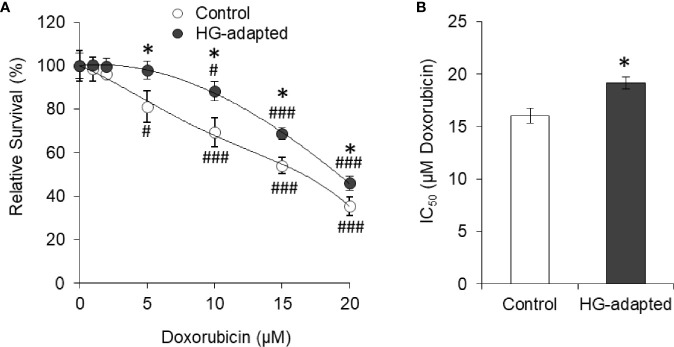
HG-adapted cells show chemoresistance. HepG2 cells were adapted in HG medium for six frequent passages (every 3 days). Cell survival of control and HG-adapted cells was estimated after incubation in medium containing increasing concentration of doxorubicin **(A)**. IC_50_ of doxorubicin was estimated for both the cells **(B)**. The values shown are Mean ± SD of three independent experiments conducted in triplicate. *p < 0.05 *vs* values of cells of control group. ^#^ *p* < 0.05, ^###^ *p* < 0.001 *vs* values of cells incubated in medium without doxorubicin.

To determine transient or persistent nature of effect, HepG2 cells were adapted in HG medium (HG-adapted) for six frequent passages (every 3 days). Control and HG-adapted cells were then incubated in medium containing increasing concentration of doxorubicin followed by estimation of cell survival by crystal violet assay. HG-adapted cells were found significantly resistant for decline in cell survival caused by doxorubicin (**Figure 3A**). IC_50_ of doxorubicin was found to be significantly higher against HG-adapted cells as compared to control (**Figure 3B**).

### Curcumin Prevents High Glucose-Induced Chemoresistance

**Figure 4:**
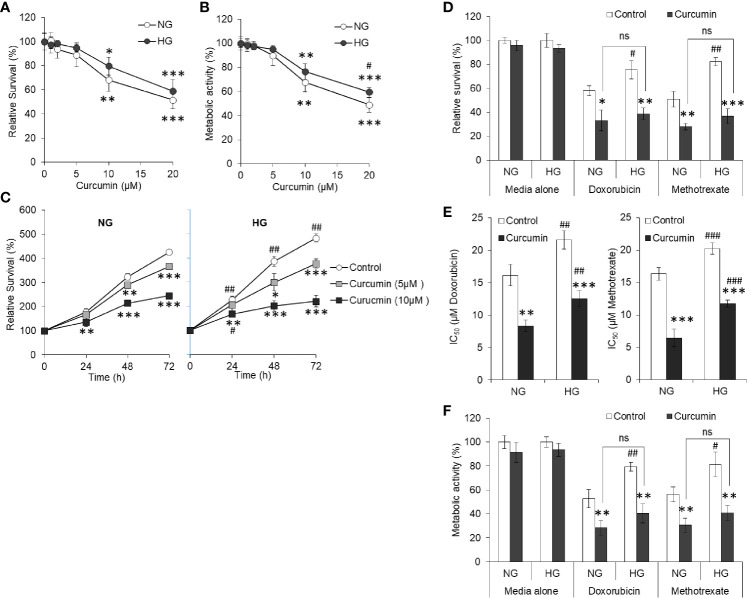
Curcumin resists the hyperglycemia-induced chemoresistance. HepG2 cells incubated in NG or HG medium with indicated concentration of curcumin for 24 h followed by estimation of cell survival **(A)** and metabolic activity **(B)**. In time kinetic study, cell survival was estimated after indicated incubation period in NG or HG medium alone or containing 5 or 10 µM curcumin **(C)**. Cells were incubated in NG or HG medium containing either 5 µM curcumin, anticancer drugs (15 µM), or their combinations for 24 h followed by estimation of cell survival **(D)**, calculation of IC_50_ **(E)**, and estimation of metabolic activity **(F)**. The values shown are Mean ± SD of three independent experiments conducted in triplicate. *p < 0.05, **p < 0.01, ***p < 0.001 *vs* values of cells of control group not treated with curcumin. ^#^ *p* < 0.05, ^##^ *p* < 0.01, ^###^ *p* < 0.001 *vs* values of cells incubated in NG medium. ns, not significant.

HepG2 (1×10^5^/ml) cells were incubated in NG or HG medium containing the indicated concentration of curcumin followed by estimation of cell survival and metabolic activity. Results are given in **Figures 4A, B**. It was found that with increasing concentration of curcumin, cell survival of HepG2 cells decreased in both NG and HG medium. Although the decline of cell survival was less in cells incubated in NG medium, there were no statistically significant differences. Metabolic activity was also found to follow similar trend except significant difference between NG and HG groups at 20 µM curcumin concentration. Relative survival after different incubation period was also determined for HepG2 cells incubated in NG or HG medium alone or containing 5 or 10 µM curcumin (**Figure 4C**). Significantly rapid increase in survival of control cells (not treated with curcumin) was observed in HG medium as compared to NG medium. Presence of curcumin resisted the time-dependent increase of cell survival both in NG or HG medium. Nevertheless, no significant difference was observed in time-dependent increase in survival of cells incubated in NG or HG medium with curcumin. Among curcumin treated groups, the decline of cell survival was insignificant after 24 h of incubation 5 µM both in NG or HG medium. In both the NG and HG medium, 5 µM curcumin was sublethal as it was found to cause no significant decline in survival of HepG2 cells after 24h. Therefore, this concentration and time of incubation were used in subsequent chemosensitization experiments, otherwise stated.

To evaluate the effect of curcumin on hyperglycemia-driven chemoresistance in tumor cells, HepG2 cells were incubated in NG or HG medium alone or containing doxorubicin or methotrexate at 15 µM (approximate to their IC_50_ in NG medium) in the absence or presence of a sublethal concentration of curcumin. Survival, IC_50_, and metabolic activity of HepG2 cells were estimated as per the standard method given in *Materials and Methods*. As indicated in **Figure 4D**, a significant decline in the survival of HepG2 cells treated with anticancer drugs along with curcumin was observed as compared to those treated with anticancer drugs alone both in NG and HG medium, indicating cooperation between curcumin and anticancer drugs. The difference in the level of cell survivals in NG and HG medium containing anticancer drugs in presence of a sublethal concentration of curcumin was found to be insignificant. Curcumin was also found to significantly decrease the IC_50_ of anticancer drugs (doxorubicin and methotrexate) in both the mediums either containing NG or HG levels (**Figure 4E**). Metabolic activity of HepG2 cells followed the trend similar to observation of cell survival with same treatment (**Figure 4F**).

### Curcumin Cooperates With the Anticancer Drug Against High Glucose to Induce Cell Death

**Figure 5:**
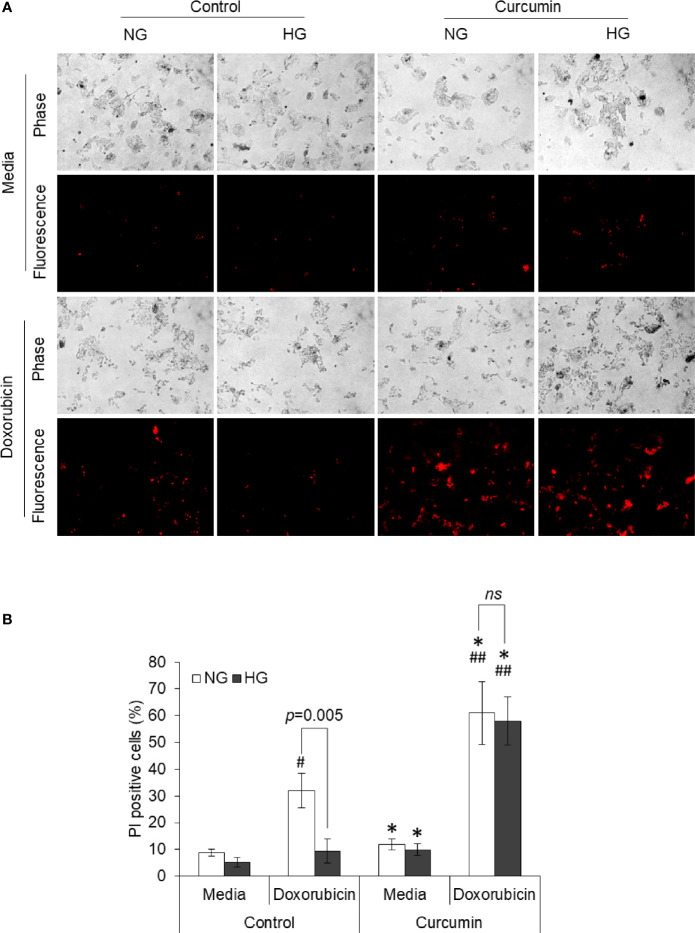
Curcumin upraises the inhibited induction of cell death by doxorubicin in HG medium. HepG2 cells incubated in NG or HG medium with and without curcumin or doxorubicin (10 µM) for 24 h. PI staining was done, and cells were observed under fluorescence microscopy to detect dead cell population **(A)**. Fluorescing dead cell percentage was enumerated **(B)**. Photomicrographs shown are representative of three experiments conducted independently. The values shown are Mean ± SD of three independent experiments conducted in triplicate. *p < 0.05 *vs* values of cells of control group not treated with curcumin. ^#^ *p* < 0.05, ^##^ *p* < 0.01 *vs* values of cells not treated with doxorubicin. ns, not significant.

Dead cell population in HepG2 cells treated with doxorubicin in NG or HG medium in absence or presence of curcumin was discriminated from live cells using PI uptake assay. Cells with red fluorescence were enumerated. Representative photomicrographs and enumerated dead cell population are given in **Figure 5**. Doxorubicin was found to augment the dead cell population in HepG2 cells incubated in NG medium; however, there was a lower percentage of PI-positive cells in the HG group of cells treated with doxorubicin. When cells are treated with doxorubicin in the presence of curcumin, a significant increase in fluorescent cells was observed both in medium containing NG or HG level, and there was no significant difference in percent PI-positive cells.

### Curcumin Inhibits the Glucose Consumption by HepG2 Cells

**Figure 6:**
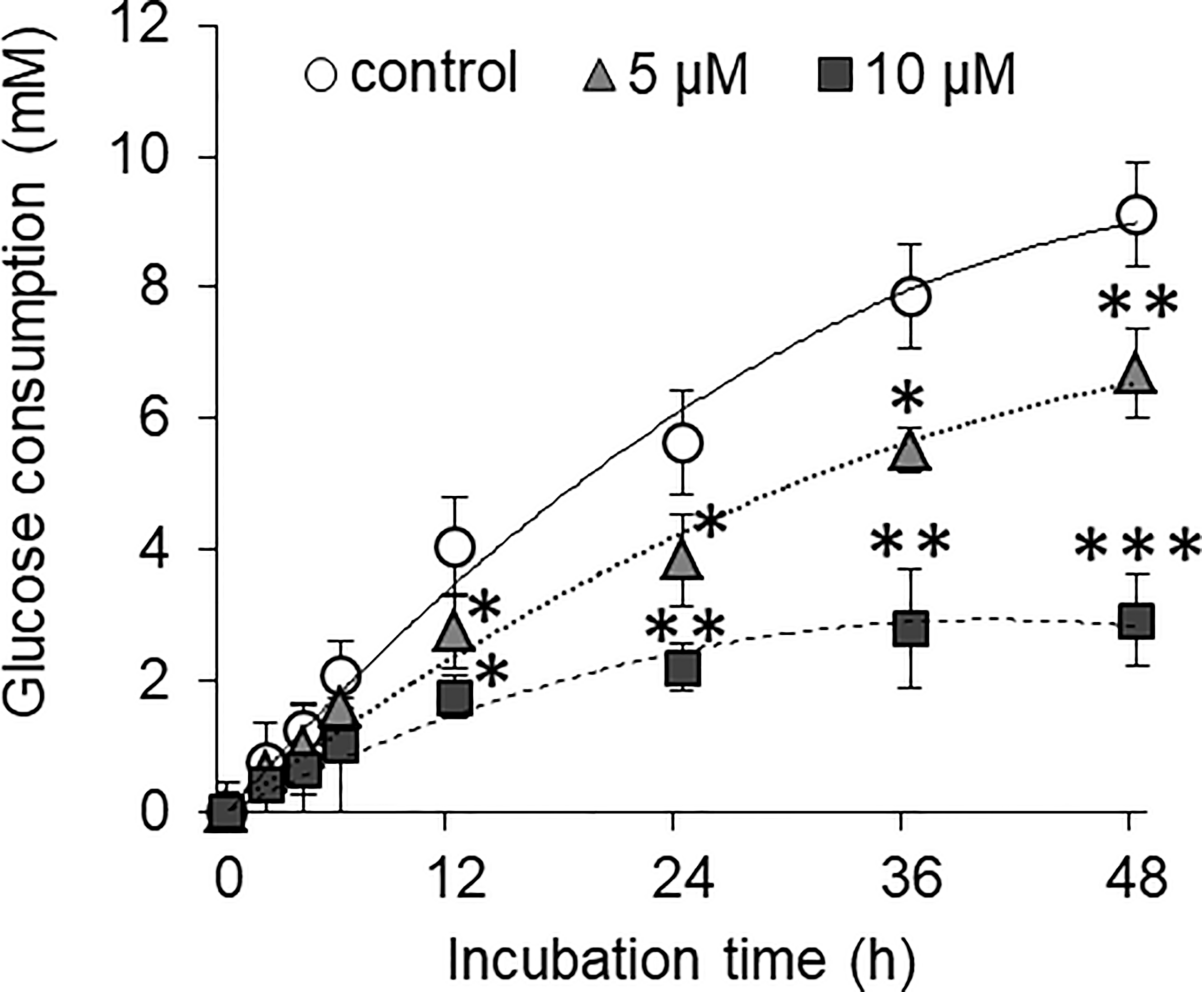
Curcumin inhibits the glucose consumption by HepG2 cells. Cancer cells were treated with curcumin at 5 or 10 µM concentrations for 12 h. Cells were then washed and plated at equal cell density in medium containing standard amount of glucose. Cell-free supernatants were collected at indicated time interval, and the amount of remaining glucose present was estimated. Difference between amount of glucose in culture supernatant and uninoculated medium was calculated to get amount of glucose consumed. The values shown are Mean ± SD of three independent experiments conducted in triplicate. *p < 0.05, **p < 0.01, ***p < 0.001 *vs* values of cells of control group not treated with curcumin.

HepG2 cells were incubated in a medium containing normal glucose level alone or in presence of either 5 or 10 µM concentration of curcumin. Cell-free supernatants were harvested at the regular time interval as indicated. The concentration of remaining glucose was estimated and was subtracted from the initial glucose concentration to determine the amount of glucose consumed. Results are given in **Figure 6**. The amount of consumed glucose was found to be increasing with the time of incubation. Both 5 and 10 µM concentrations of curcumin inhibited the glucose consumption by HepG2 cells. Glucose consumption was found to be significantly lower by HepG2 cells incubated in a curcumin-containing medium for 12 h or more. The difference in glucose consumption was found more significant when cells were incubated in a medium containing 10 µM of curcumin for 24 h or more.

### Curcumin Resists the Alteration in the Extracellular Milieu by the High Level of Glucose

To evaluate the ability of curcumin on the high level of glucose-mediated alterations in extracellular pH and lactate level, HepG2 (1×10^5^/ml) cells were incubated in medium containing a normal or high concentration of glucose in the absence or presence of curcumin (5 µM), and cell-free culture supernatants were collected. The pH of culture supernatants was estimated using a pH meter, and lactate levels were estimated as per the method given in *Materials and Methods*. Results are given in **Table 2**. The culture supernatant of cells incubated in the HG medium was found to have significantly acidic (lower) pH as compared to those incubated in the NG medium. Curcumin treatment was found to resist the lowering of pH of culture supernatant both in NG as well in HG medium. The lactate level in the culture supernatant was found to be significantly higher for the cells incubated in the HG medium. Curcumin treatment decreases the lactate level in the culture supernatant of cells incubated either in an NG or HG-containing medium. No significant difference was found in the lactate level in the culture supernatant of cells treated with curcumin either in NG or HG medium.

**Table 2:** Curcumin resisted the high glucose-induced alterations in extracellular lactate, NO, and pH level.

Parameters	Control		Curcumin	
	NG	HG	NG	HG
pH	7.09 ± 0.069	6.93 ± 0.094^##^	7.20 ± 0.023**	7.08 ± 0.91*^#^
Lactate (mM)	3.51 ± 0.20	.69 ± 0.54^#^	2.69 ± 0.47*	3.61 ± 0.56*
NO (µM/10^6^Cells)	32.77 ± 2.44	26.73 ± 2.59^##^	37.38 ± 1.61**	35.44 ± 2.70***

Cell-free culture supernatant was collected from HepG2 cells incubated in NG or HG medium with or without curcumin at sublethal concentration. pH level along with concentration of lactate and NO was determined. The values shown are Mean ± SD of three independent experiments conducted in triplicate. *p < 0.05, **p < 0.01, ***p < 0.001 vs values of cells of control group not treated with curcumin. ^#^p < 0.05, ^##^p < 0.01 vs values of cells incubated in NG medium.

Nitric oxide level was also estimated in the culture supernatant of HepG2 cells treated with curcumin in a medium either containing a normal or high level of glucose. Results are given in **Table 2**. Culture supernatant of cells incubated in the HG medium had a significantly lower level of nitric oxide. Curcumin was found to significantly augment the level of nitric oxide in culture supernatants of the cells incubated in NG and HG medium. There was no significant difference observed in the culture supernatant NO level of NG or HG groups treated with a sublethal concentration of curcumin.

### Curcumin Favors ROS Induction by ACD

**Figure 7:**
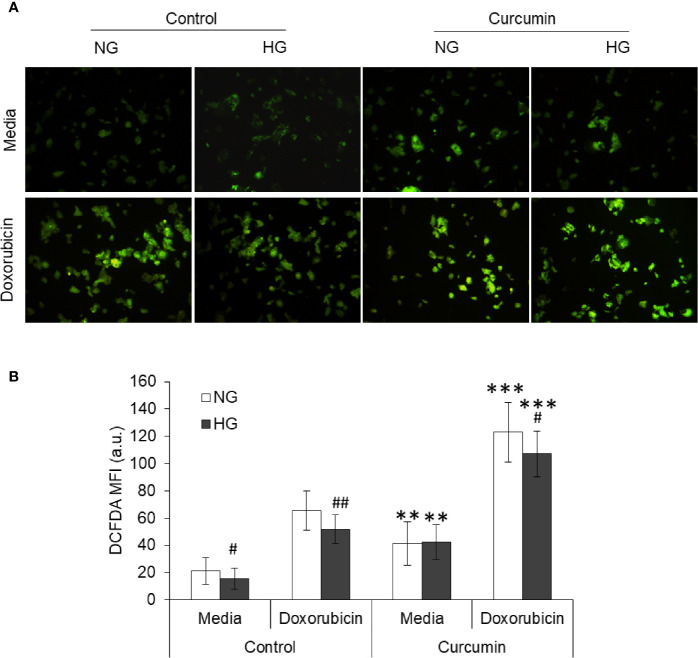
Curcumin favors the ROS production in HepG2 cells in normal as well as hyperglycemic conditions. Using DCFDA fluorescence, ROS production was detected in cancer cells treated with curcumin, or doxorubicin, or their combinations in NG or HG medium for 24 h. Photomicrograph of DCFDA stained cells were captured **(A)**. Mean fluorescence intensity (MFI) of cells was determined using ImageJ software **(B)**. Photomicrographs shown are representative of three experiments conducted independently. The values shown are Mean ± SD of three independent experiments conducted in triplicate. **p < 0.01, ***p < 0.001 *vs* values of cells of control group not treated with curcumin. ^#^ *p* < 0.05, ^##^ *p* < 0.01 *vs* values of cells incubated in NG medium.

Tumor cells treated with doxorubicin in NG or HG medium with and without sublethal concentration of curcumin were processed for detection of ROS generation using DCFDA and observed under a fluorescent microscope. Images of fluorescence were processed using ImageJ software for the determination of mean fluorescence intensity (MFI). Results are given in **Figure 7**. Doxorubicin treatment significantly augmented the level of ROS in the cells incubated either in NG or HG medium. However, the ROS level was found to be significantly lower in doxorubicin-treated cells incubated in HG medium as compared to those incubated in NG medium. Treatment of cells with doxorubicin in presence of curcumin was found to cooperatively augment the ROS level in cells of both NG and HG groups.

### Curcumin Pretreatment Resists the Effect of High Glucose

**Figure 8:**
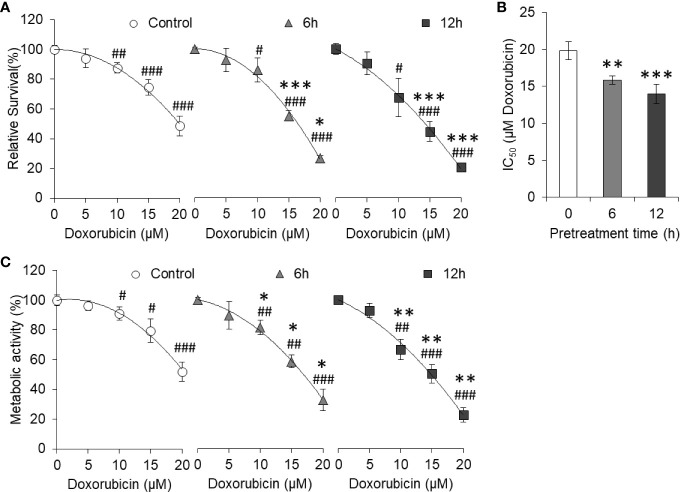
Curcumin pretreatment prevents high glucose-induced chemoresistance. HepG2 cells treated for 6 or 12 h with curcumin were washed and plated at equal density in HG medium containing increasing concentration of doxorubicin. Cell survival was estimated using standard crystal violet assay **(A)** and calculation of IC_50_ **(B)**. Metabolic activity of cells was also estimated using MTT assay **(C)**. The values shown are Mean ± SD of three independent experiments conducted in triplicate. *p < 0.05, **p < 0.01, ***p < 0.001 *vs* values of cells not pretreated with curcumin. ^#^ *p* < 0.05, ^##^ *p* < 0.01, ^###^ *p* < 0.001 *vs* values of cells of not treated with doxorubicin.

To evaluate whether pretreatment of curcumin can have any effect on hyperglycemia-mediated chemoresistance, HepG2 cells (1×10^5^/ml) were incubated in sublethal (5 µM) curcumin containing medium for a time indicated in the results. Cells were then washed and cultured in a medium containing 25 mM glucose (HG) with indicated concentrations of doxorubicin. After 24 h of incubation, cell survival, IC_50_, and metabolic activity were estimated as per the standard method described in *Materials and Methods*. It was found that doxorubicin was able to inhibit the cell survival of HepG2 cells in a concentration-dependent manner in control as well as curcumin pretreated cells (**Figure 8A**). However, in pretreated cells, survival of doxorubicin-treated HepG2 was significantly lower as compared to cells now exposed to curcumin. Differences in the survival of doxorubicin-treated cells between control and curcumin pretreated groups were found to be significantly higher in the group pretreated for longer (12 h) duration. Curcumin pretreatment was found to significantly decrease the IC_50_ of doxorubicin against tumor cells incubated in an HG medium in a pretreatment time-dependent manner (**Figure 8B**). Doxorubicin-mediated decline in metabolic activity was also observed in control as well as curcumin-pretreated HepG2 cells (**Figure 8C**). The rate of decline in metabolic activity by doxorubicin was significantly higher in curcumin pretreated cells as compared to control.

### Curcumin Alters High Glucose-Modulated Nuclear Dynamics

**Figure 9:**
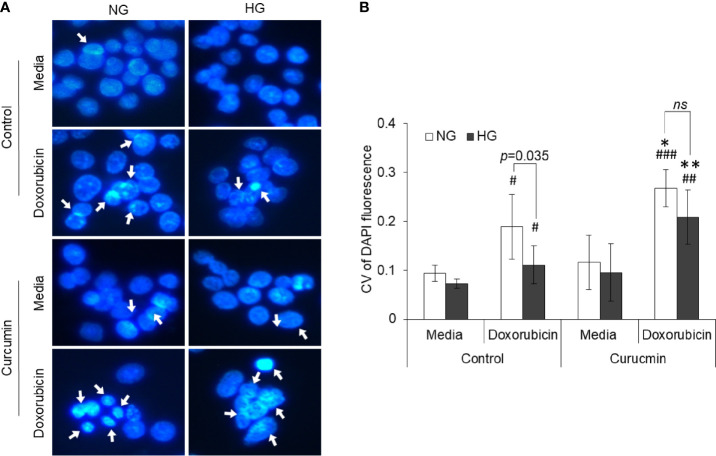
Counteracting action of curcumin on inhibition of doxorubicin-induced chromatin condensation in HG medium. HepG2 cells treated doxorubicin, curcumin, or their combinations in NG or HG medium were processed for detection of chromatin condensation through DAPI staining. DAPI-stained cells were observed under fluorescence microscope, and images were captured **(A)**. Arrowheads indicate chromatin condensation. To determine the chromatin compactness, DAPI-stained cells after Sobel edge detection transformation were analyzed with ImageJ software for all the pixels in each nucleus. Coefficient of variation was determined for all cells **(B)**. The values shown are Mean ± SD of three independent experiments conducted in triplicate. Photomicrographs shown are representative of three experiments conducted independently. *p < 0.05, **p < 0.01 *vs* values of cells of control groups not treated with curcumin. ^#^ *p* < 0.05, ^##^ *p* < 0.01, ^###^ *p* < 0.001 *vs* values of cells incubated in medium without doxorubicin. ns, not significant.

The effect of HG and curcumin treatment on nuclear dynamics was detected by observing the degree of chromatin condensation using DAPI staining. Cells were incubated in NG or HG medium containing doxorubicin, curcumin, or their combination for 24 h. Cells were then stained with DAPI and observed under a fluorescent microscope. Representation images are given in **Figure 9A**. Photomicrographs were then processed for Sobel edge detection and analyzed for the coefficient of variation (CV) of DAPI fluorescence to determine chromatin condensation. Results are given in **Figure 9B**. Chromatin condensation was found to be augmented in the doxorubicin-treated group as compared to the control group incubated in an NG medium. Doxorubicin was also found to augment the chromatin condensation in cells incubated in HG medium; however, the level of condensation was significantly lower as compared to the NG group. The presence of curcumin during treatment both in NG or HG medium conjoins the doxorubicin in elevating the chromatin condensation in HepG2 cells.

### Curcumin Favors Drug Accumulation in Cancer Cells

**Figure 10:**
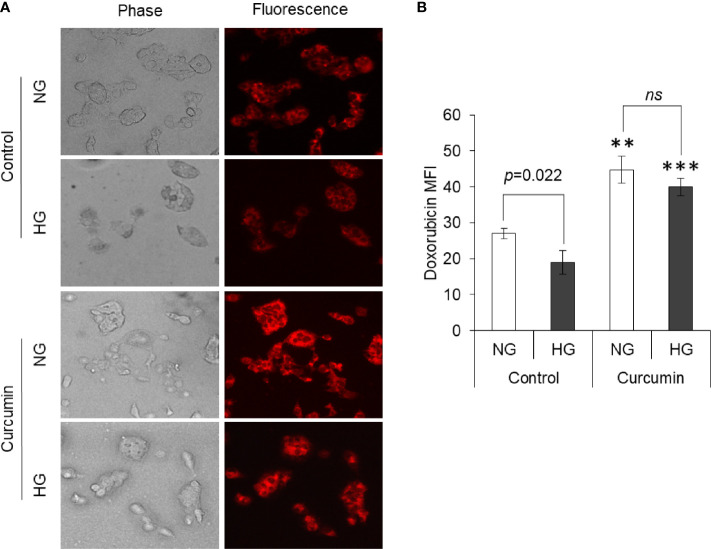
Curcumin promotes intracellular accumulation of drugs. Drug uptake by cells incubated in NG or HG medium alone or containing curcumin was detected. Doxorubicin fluorescence was detected using fluorescence microscope **(A)**. Mean fluorescence intensity (MFI) of cells was determined **(B)**. Photomicrographs shown are representative of three experiments conducted independently. The values shown are Mean ± SD of three independent experiments conducted in triplicate. **p < 0.01, ***p < 0.001 *vs* values of cells of control group not treated with curcumin. ns, not significant.

The effect of curcumin and hyperglycemia on the accumulation of anticancer drugs was evaluated by incubating the HepG2 cells (1×10^5^/ml) in NG or HG medium alone or containing the sublethal concentration of curcumin. Cells were then washed and incubated in a medium containing doxorubicin for drug uptake assay as indicated in *Materials and Methods*. After washing, cells were observed under a fluorescent microscope, and images were captured. MFI of doxorubicin in cells was estimated using ImageJ software. Results are given in **Figure 10**. Cells incubated in a medium having hyperglycemic conditions have a significantly lower level of doxorubicin fluorescence. The curcumin was found to significantly heighten the doxorubicin fluorescence in cells incubated in a medium with either normal or high levels of glucose. There was no statistically significant difference observed in doxorubicin fluorescence of cells incubated in HG or NG medium containing a sublethal concentration of curcumin.

### Curcumin Modulates mRNA Expression Both in NG as Well as HG Medium

**Figure 11:**
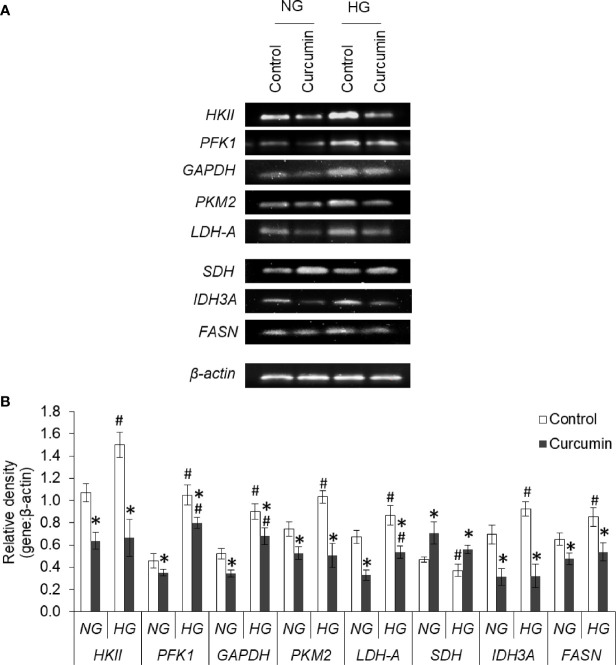
Curcumin modulates the expression of metabolic enzymes. Cells incubated in NG or HG medium with and without curcumin were processed for RT-PCR analysis for gene expression of metabolic enzymes. The amplified DNA were resolved on Agarose gel, bands were visualized, and images of bands were captured **(A)**. Densitometric scan of bands were done, and relative intensity of bands against β-actin was calculated **(B)**. Bands shown are representative of three experiments conducted independently. The values shown are Mean ± SD of three independent experiments conducted in triplicate. *p < 0.05 *vs* values of cells of control group. ^#^ *p* < 0.05 values of cells incubated in NG medium.

The effect of the high level of glucose and curcumin on the expression profile of various metabolic enzymes was evaluated by determining the mRNA level using RT-PCR analysis. Representative images of bands are given in **Figure 11A**. A densitometric scan of the bands obtained after RT-PCR and gel electrophoresis was also done, and the relative intensity of bands of genes against bands of β-actin are given in **Figure 11B**. It was found that HG favors the augmented expression of most of the glycolytic enzymes (HKII, PFK1, GAPDH, PKM2, and LDH-A), IDH3A, and fatty acid synthase (FASN). In contrast, the expression level of SDH was found to be significantly lower in cells incubated in a medium containing a high concentration of glucose. The sublethal concentration of curcumin was found to significantly inhibit the mRNA expression of HKII, PFK1, GAPDH, PKM2, and LDH-A in the cells incubated in a medium containing either normal or high concentrations of glucose. A similar trend was observed with the expression of IDH3A and FASN. For the expression of SDH, curcumin treatment augmented the mRNA level in cells of either NG or HG groups.

**Figure 12:**
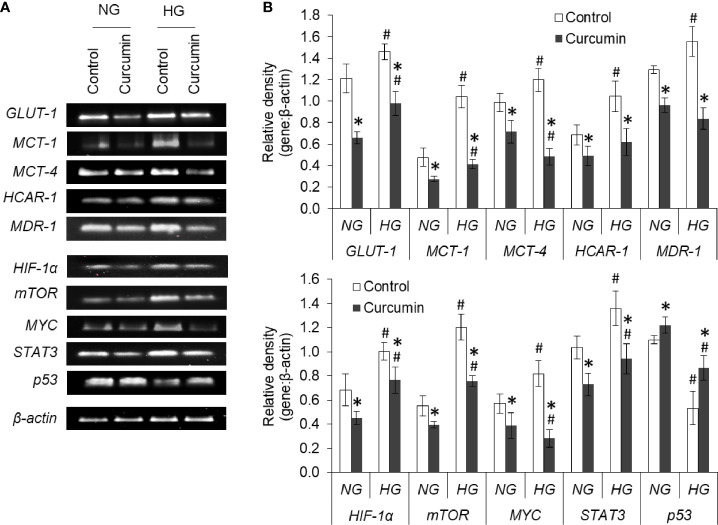
Curcumin modulates the gene expression of regulators of metabolic and chemoresistance phenotype of cancer cells. Cells incubated in NG or HG medium with and without curcumin were processed for RT-PCR analysis for gene expression of regulators of metabolic and chemoresistance phenotype as indicated. The amplified DNA were resolved on Agarose gel, bands were visualized, and images of bands were captured **(A)**. Densitometric scan of bands were done, and relative intensity of bands against β-actin was calculated **(B)**. Bands shown are representative of three experiments conducted independently. The values shown are Mean ± SD of three independent experiments conducted in triplicate. *p < 0.05 *vs* values of cells of control group. ^#^ *p* < 0.05 values of cells incubated in NG medium.

The effect of the high level of glucose and curcumin on the expression level of mRNA of various transporters, receptors, and transcription factors was also evaluated. The results are given in **Figures 12A, B**. Hyperglycemia was found to augment the expression of GLUT-1, MCT-1, MCT-4, HCAR-1, and MDR-1. Curcumin was found to oppose this HG level-mediated alteration in mRNA expression of cell surface molecules. Among the transcription factors and signaling molecules, an elevated level of glucose in the culture medium favors the boosted expression of HIF-1α, mTOR, MYC, and STAT3. Curcumin treatment significantly decreases the mRNA level of these molecules in cells incubated both in NG or HG medium. HG medium cells were found to have a significantly lower level of p53 mRNA which was found to be elevated with treatment with curcumin.

### Curcumin Counters the HG-Modulated Protein Expression

**Figure 13:**
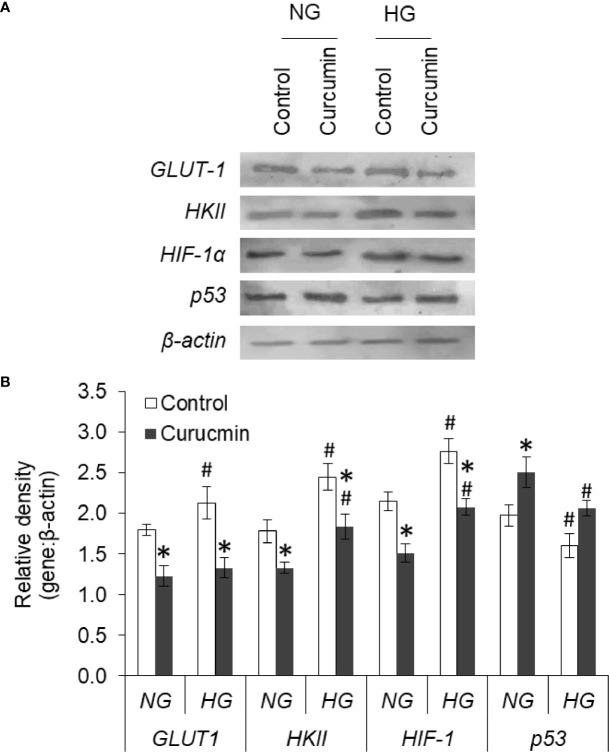
Curcumin modulates the protein expression of GLUT-1, HKII, HIF-1α, and p53. HepG2 cells were incubated in NG or HG medium with and without curcumin and GLUT-1, HKII, HIF-1α, and p53 expression was detected by western blot analysis **(A)**. Densitometric scan of bands were done, and relative intensity of bands against β-actin was calculated **(B)**. Bands shown are representative of three experiments conducted independently. The values shown are Mean ± SD of three independent experiments conducted in triplicate. *p < 0.05 *vs* values of cells of control group. ^#^ *p* < 0.05 values of cells incubated in NG medium.

Expression levels of GLUT1, HKII, HIF-1α, and p53 were found to alter in HG medium (**Figure 13**). The expression level of GLUT1, HKII, and HIF-1α was found significantly higher in HepG2 cells incubated in HG medium as compared to NG medium. Curcumin treatment was able to significantly decrease the expression of GLUT1, HKII, and HIF-1α in HepG2 cells either incubated in NG or HG medium. The protein expression level of p53 was found to significantly decline in cell incubated in HG medium. Significant augmentations in protein expression level of p53 were observed in curcumin-treated cells, both in NG or HG medium group, as compared to their respective controls.

## Discussion

The present investigation was taken up to explore the ability of curcumin on high glucose-induced chemoresistance. High level of glucose favored the proliferation along with a significant decline in the number of dead cells; and triggered the resistance and drug-induced cell death in hepatic carcinoma cells. Hyperglycemic conditions incept the chemoresistant phenotype in a variety of malignant cells (4, 6, 8, 9, 37). Modulation of various molecular regulators of cell survival was reported to play a critical role in high glucose-triggered chemoresistance (6, 24, 37). A significant decline in the expression of p53 was observed in HepG2 cells treated with high glucose level. p53 is known to mediate the induction of apoptosis in a variety of cancer cells including those of hepatocellular origin (38). Interestingly, high glucose was not observed to confer resistance against the direct cytotoxic activity of curcumin. Moreover, curcumin was found to avert the high glucose-mediated resistant behavior of HepG2 cells. An increased expression of p53 in curcumin-treated cells can be suggestive of susceptibility towards cytotoxic action of anticancer drugs (38). Although the ability of curcumin to circumvent the chemoresistance is already proven (14, 18, 19, 22, 25), its potential against high glucose-mediated resistant behavior in HepG2 cells are being first time reported. However, it will be noteworthy to mention here that curcumin has therapeutic benefits in hyperglycemia-associated pathological manifestations and through NF-κB inhibition (16, 23). NF-κB is also implemented in the onset of chemoresistance (39); and curcumin can suppress the NF-κB signaling (40). Both in simultaneous and pretreatment experiments, curcumin prevented the high glucose-induced chemoresistance. This suggested a cooperative influence between curcumin and chemotherapeutic drugs, and changes in cellular physiology opposing inception of chemoresistance.

Curcumin was found to avert the high glucose-triggered changes in the extracellular milieu. The enhanced level of nitric oxide was observed in curcumin treated groups, both in NG or HG medium. Both pro- and antitumor effects of nitric oxide level are reported (41), which are largely affected by factors including type of tissue, concentration level, cell sensitivity, tumor microenvironment, and hypoxia/re-oxygenation level (41, 42). In liver cancer, elevated level of nitric oxide is linked with growth arrest and apoptosis along with chemosensitization through modulated levels of p53 and HIF-1α (42). Curcumin-mediated increased extracellular pH and decreased lactate can also be correlated with augmented sensitivity towards anticancer drugs (5, 6). Previously, lactate was reported to enhance the chemoresistance in cancer cells (14, 43). A decline in LDH level can be suggested to decrease the generation of lactate by cancer cells treated with curcumin. A curcumin-mediated decline in expression of MCT-1 and MCT-4 may also contribute to reduction in extracellular lactate level. Similarly, suppressed GLUT-1 expression by curcumin can cause a decrease in glucose consumption. Similar to previous investigations (17, 26), a decline in glucose consumption and production of lactate by curcumin-treated cancer cells was observed, indicating the repression of glycolytic Warburg phenotype.

High glucose treatment was able to repress the production of ROS in HepG2 cells treated with doxorubicin. Repressed production of ROS in high glucose treatment is in line with findings of previous investigation (4). ROS has a dual role to play in cancer biology; and its low level upkeeps the survival of cancer stem cells, which correlates with chemoresistance (44, 45). Curcumin was able to augment the ROS production by doxorubicin even in the hyperglycemic condition. Although curcumin is known for free radical scavenging activity, opposing effect on ROS production has been reported (46, 47). Curcumin has been previously shown to synergize the augmentation of ROS level by anticancer drugs (47). SDH and IDH3A have been implemented in the regulation of ROS generation in malignant pathologies (48), chemoresistance (49), glucose uptake (50), and invasion (51). Curcumin was able to induce SDH expression and repress the IDH3a in HepG2 cells both in a normal or elevated level of glucose. Such changes in SDH and IDH3a levels can bring a reduction in the succinate accumulation and hindering the succinate-HIF-1α axis (52, 53). The augmented expression of HIF-1α in high glucose conditions was resisted by curcumin. HIF-1α is known for metabolic regulation in malignant cells, their hyperglycolytic behavior, and the onset of chemoresistance (54). HIF-1 exerts protumor effects through the upregulated expression of enzymes and transporters favoring the hyperglycolytic and therapy-resistant phenotype (1, 39, 49, 53, 54).

Augmented expression of glycolytic enzymes and transporters are correlated with hyperproliferative and chemoresistant behavior in cancer cells (5, 24, 54–56). An elevated level of GLUT-1 ensures high uptake to meet the demand for glucose for accelerated glycolysis in aggressive cancer cells (54, 56). Expression of GLUT-1 was augmentation in high glucose–exposed cells. An augmented expression of HKII in HepG2 cells in hyperglycemic condition was also observed. HKII also contributes to the prevention of apoptotic death in malignant cells and favor chemoresistance (57, 58). Along with inhibition of GLUT-1 and HKII, curcumin also moderated the high glucose-induced expression of PFK1, GAPDH, and PKM2. The observed decrease in PKM2 expression is in line with a previous report where curcumin mediates a fall in Warburg phenotype through downregulated PKM2 (26). PKM2 also correlates with chemoresistance through HIF-1 and serves as co-activator for the transcription factor (26, 57, 58). Augmented expression of GAPDH is linked with autophagy in cancer cells (59). Autophagy serves as important machinery during oncogenic transformation as well as the onset of chemoresistance (1, 59). Therefore curcumin-mediated repression on GAPDH expression can be sought as molecular modulation leading to impeded glycolysis, and chemosensitization. A decreased expression of PFK1 by curcumin in HepG2 cells can also be expected to thwart the hyperactive glycolytic pathway, augmented proliferation, and chemoresistant behavior (58, 60). siRNA-mediated silencing of PFK1 was demonstrated to suppresses glycolysis and increases the sensitivity towards therapeutic interventions (60). Tumor acidosis in hyperglycolytic cancer cells enhances the fatty acid synthesis through activation of FASN (5). FASN expression can be upregulated by HIF-1 and contribute to the initiation and progression of malignancies (54); and its targeting can avert the chemoresistance (61). Alleviation of FASN expression in high glucose condition by curcumin can be expected to chemosensitize cancer cells.

Aggressive behavior and chemoresistant phenotype of cancer cells also correlate with unique nuclear mechanics (14, 43). As assessed by DAPI staining, high glucose level prevented this drug-induced nuclear condensation in HepG2 cells. However, curcumin cooperated with the anticancer drug in enhancing the condensation of nuclear content of HepG2 cells, both in normal and high glucose conditions. Curcumin also repressed the high glucose-enhanced expression of both mTOR and STAT3; both of these have implication in nuclear dynamics (62). Curcumin also inhibited the elevated expression of MYC in HepG2 cells exposed to high glucose condition. The transcription factor MYC is considered to favor the oncogenic transformation and stimulate the Warburg phenotype along with activation of HIF-1 and loss of p53 (63). High glucose upregulated the level of MDR-1, which can be expected the intracellular accumulation of anticancer drugs (1, 2, 49). Interestingly, reduced accumulation of doxorubicin was recorded in cells cultured in high glucose media. Curcumin-mediated inhibition of MDR-1 expression can be suggested as critical event leading to retention of anticancer drug in cellular interior. Curcumin averted the high glucose-triggered augmented expression of HCAR-1 in HepG2 cells. HACR-1 can affect the compactness of chromatin and upon stimulation with lactate can induce chemoresistance in cancer cells *via* upregulation of MDR-1 (14, 32, 43). Therefore, it can be suggested that curcumin acted through multiple dimensions to repress the expression of molecules favoring chemoresistance. Inhibited HACR-1 and limited availability of its ligand lactate caused by curcumin culminated to decreased expression of drug efflux transporters.

The findings presented in this investigation for the first time indicate that curcumin has the ability to avert high glucose-induced chemoresistance in cancer cells. Various aspects of the underlying mechanism were also explored. Curcumin mediated the amputation of chemoresistance by repressing the hyperglycolytic behavior of malignant cells *via* modulated expression of metabolic enzymes (HKII, PFK1, GAPDH, PKM2, LDH, SDH, IDH, and FASN), transporters (GLUT-1, MCT-1, and MCT-4), and their regulators. Along altered constitution of extracellular milieu, these molecular changes culminated into improved drug accumulation, chromatin condensation, and induction of cell death. Molecular alterations in the expression level of transcription factors (p53, HIF-1α, MYC), drug efflux pumps (MDR-1), and their regulators (HCAR-1, mTOR, and STAT3) can be suggested as the underlying molecular mechanism. This investigation contributed to the understanding of the anticancer ability of curcumin through the prevention of chemoresistance in hyperglycemic conditions along with underlying mechanisms. The demonstrated potential of curcumin against high glucose-induced chemoresistance will have implementations in clinical management of malignancies in diabetic patients.

## Data Availability Statement

The raw data supporting the conclusions of this article will be made available by the authors, without undue reservation.

## Author Contributions

NV, AK, and DS conceptualized the study and designed the experiments. VS performed the experiments presented in the study. VS, AM, YR, AK, VC, DS, and NV did the data analysis. VS, DS, and NV wrote the manuscript. All authors contributed to the article and approved the submitted version.

## Conflict of Interest

The authors declare that the research was conducted in the absence of any commercial or financial relationships that could be construed as a potential conflict of interest.

## Publisher’s Note

All claims expressed in this article are solely those of the authors and do not necessarily represent those of their affiliated organizations, or those of the publisher, the editors and the reviewers. Any product that may be evaluated in this article, or claim that may be made by its manufacturer, is not guaranteed or endorsed by the publisher.

## Supplementary Material

Supplementary Figure 1 Invariable gene expression of β-actin. Cells were analyzed for gene expression of metabolic enzymes under different treatments. Intensities of β-actin bands after RT-PCR amplification were determined and plotted against amount of template along with calculation of coefficient of correlation.

Supplementary Figure 2 Invariable β-actin gene expression. Cells were analyzed for gene expression of regulators of metabolic and chemoresistance phenotype of HepG2 cells under different treatments. Intensities of β-actin bands after RT-PCR amplification were determined and plotted against amount of template along with calculation of coefficient of correlation.

Supplementary Figure 3 High glucose condition resisted anticancer drug-induced cell death in HepG2 cells. Dead and live population were enumerated in HepG2 cells incubated in NG or HG medium with increasing concentration of anticancer drugs. Ratio of dead and live cells was calculated for cell treated with doxorubicin **(A)** or methotrexate **(B)**. The values shown are Mean ± SD of three independent experiments conducted in triplicate. **p* < 0.05, ***p* < 0.01 *vs* values of cells incubated in NG medium. ^#^ *p* < 0.05, ^##^ *p* < 0.01, ^###^ *p* < 0.001 *vs* values of cells incubated in medium without anticancer drugs.
