# Supplementary figures and images for: Counteracting Action of Curcumin on High Glucose-Induced Chemoresistance in Hepatic Carcinoma Cells
Source: Front Oncol. 2021 Oct 6;11:738961. doi: 10.3389/fonc.2021.738961 (PMC8526934; doi:10.3389/fonc.2021.738961)

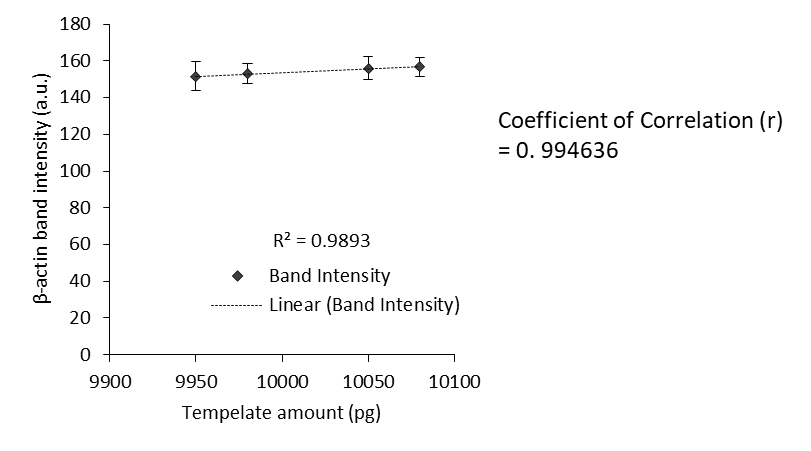

Supplement: Supplementary Figure 1 — Invariable gene expression of β-actin. Cells were analyzed for gene expression of metabolic enzymes under different treatments. Intensities of β-actin bands after RT-PCR amplification were determined and plotted against amount of template along with calculation of coefficient of correlation. [file Image_1.tif]

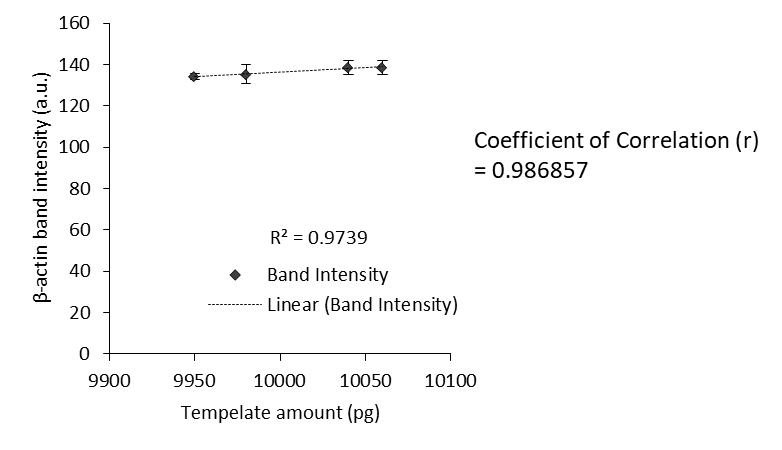

Supplement: Supplementary Figure 2 — Invariable β-actin gene expression. Cells were analyzed for gene expression of regulators of metabolic and chemoresistance phenotype of HepG2 cells under different treatments. Intensities of β-actin bands after RT-PCR amplification were determined and plotted against amount of template along with calculation of coefficient of correlation. [file Image_2.tif]

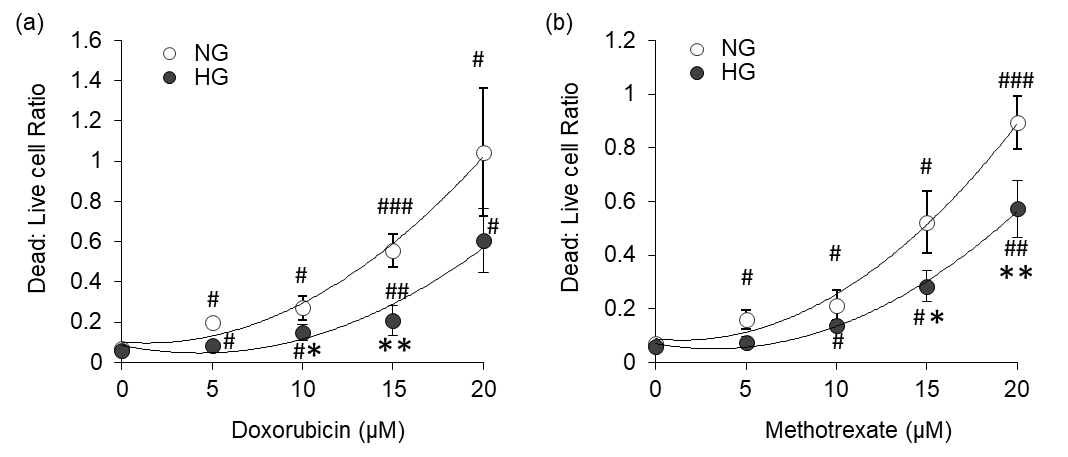

Supplement: Supplementary Figure 3 — High glucose condition resisted anticancer drug-induced cell death in HepG2 cells. Dead and live population were enumerated in HepG2 cells incubated in NG or HG medium with increasing concentration of anticancer drugs. Ratio of dead and live cells was calculated for cell treated with doxorubicin (A) or methotrexate (B). The values shown are Mean ± SD of three independent experiments conducted in triplicate. *p < 0.05, **p < 0.01 vs values of cells incubated in NG medium. # p < 0.05, ## p < 0.01, ### p < 0.001 vs values of cells incubated in medium without anticancer drugs. [file Image_3.tif]
